# Linear and Nonlinear Behaviors of the Photoreceptor Coupled Network

**DOI:** 10.1523/JNEUROSCI.1433-23.2024

**Published:** 2024-02-29

**Authors:** Ji-Jie Pang, Xiaolong Jiang, Samuel M. Wu

**Affiliations:** Departments of Ophthalmology and Neuroscience, Baylor College of Medicine, Houston, Texas 77030

**Keywords:** HCN channels, *I*_h_, junctional resistance, linear cable equation, photoreceptor coupling, rod–rod coupled network, space constant, time-to-peak, ZD7288

## Abstract

Photoreceptors are electrically coupled to one another, and the spatiotemporal properties of electrical synapses in a two-dimensional retinal network are still not well studied, because of the limitation of the single electrode or pair recording techniques which do not allow simultaneously measuring responses of multiple photoreceptors at various locations in the retina. A multiple electrode recording system is needed. In this study, we investigate the network properties of the two-dimensional rod coupled array of the salamander retina (both sexes were used) by using the newly available multiple patch electrode system that allows simultaneous recordings from up to eight cells and to determine the electrical connectivity among multiple rods. We found direct evidence that voltage signal spread in the rod–rod coupling network in the absence of *I*_h_ (mediated by HCN channels) is passive and follows the linear cable equation. Under physiological conditions, *I*_h_ shapes the network signal by progressively shortening the response time-to-peak of distant rods, compensating the time loss of signal traveling from distant rods to bipolar cell somas and facilitating synchronization of rod output signals. Under voltage-clamp conditions, current flow within the coupled rods follows Ohm’s law, supporting the idea that nonlinear behaviors of the rod network are dependent on membrane voltage. Rod–rod coupling is largely symmetrical in the 2D array, and voltage-clamp blocking the next neighboring rod largely suppresses rod signal spread into the second neighboring rod, suggesting that indirect coupling pathways play a minor role in rod–rod coupling.

## Significance Statement

This study employs the newly available multiple patch electrode technique to study the network properties of photoreceptors by simultaneously recording from multiple neurons. Unraveling how the photoreceptor coupled network shapes spatiotemporal responses, voltage gains and synaptic transfer in the outer retina is crucial for our understanding of how the brain senses the visual world. Moreover, the negative peak response velocity of the coupled rod network mediated by HCN channels synchronize rod signals converging to a bipolar cell and improves the temporal resolution of the rod output synapses, a strategy that may be used by other coupled neuronal networks in the brain.

## Introduction

Retinal circuitry is one of the most promising research platforms in the nervous system where mysteries of neural network function can be unraveled, because individual neuronal circuits therein can be selectively activated by specific natural stimuli (light of various spatiotemporal patterns, color, and contrast; Wu, 2010; Dowling, 2012). It has been shown that photoreceptors are electrically coupled (Baylor et al., 1971), and in most species photoreceptors of the same type (homotypic, such as rod–rod) are more strongly coupled than those of different types (heterotypic, such as rod–cone; Raviola and Gilula, 1973, 1975; Attwell and Wilson, 1980; Attwell et al., 1984; Hornstein et al., 2004, 2005; Li and Devries, 2004; Gao et al., 2013). Photoreceptor coupling was initially discovered by observations that receptive fields of turtle cones were substantially larger than the cells’ geometrical dimensions (Baylor et al., 1971), and it was later confirmed in many species by pair photoreceptor recordings that measure current flows from one photoreceptor to another (Attwell and Wilson, 1980; Attwell et al., 1984; Hornstein et al., 2004; Li and Devries, 2004; Zhang and Wu, 2005). A limitation of pair recordings is that it can only measure coupling strengths (junctional conductance or coupling coefficient) between two photoreceptors (Zhang and Wu, 2005), while it is difficult to determine the spatiotemporal response profiles of the photoreceptor coupled network. For example, in the spatial domain, it is important to know whether the spread of electrical signals follows the linear cable equation, indicative of passive diffusion of electrical signals (Johnston and Wu, 1994). It is also important to determine whether coupling is symmetrical or it has orientation preference in the retina, and how much heterotypic (such as rod–cone) coupling contributes to homotypic coupling. Therefore, simultaneous recordings of multiple rods and cones are needed to resolve these issues.

In the temporal domain, Hodgkin’s group showed that the kinetics of light response in distant photoreceptors to a local light spot is faster than that in nearby photoreceptors, an observation termed “inductive property” of the photoreceptor network (Detwiler et al., 1978, 1980). Later studies using pair recording techniques suggested that a hyperpolarization-activated current (*I*_h_) mediated by hyperpolarization-activated cyclic nucleotide-gated channel (HCN1 channels) in photoreceptors may be involved (Attwell and Wilson, 1980; Attwell et al., 1984; Barrow and Wu, 2009). However, due to the limitation of the single electrode or pair recording techniques, it has not been possible to directly verify this phenomenon by simultaneously measuring response kinetics of multiple photoreceptors at various locations in the retina. A multiple electrode recording system is needed to achieve these goals.

In recent years, simultaneous whole-cell recordings from 8–12 neurons have been successfully made to study synaptic interconnections among cortical neurons (Jiang et al., 2015; Cadwell et al., 2017, 2020). In this project, we adopted this new recording system for the first time to study retinal circuitry, specifically on the spatiotemporal properties of the coupled photoreceptor network. This approach is capable of revealing important new insights on how multicellular circuits process visual information, not obtainable by single or dual electrode recording techniques. To determine how intermediate neurons connecting two cells in the network function, we adopted a novel “voltage-clamp block” method to block voltage signal transmission from cell A to cell C by voltage clamping the intermediate cell B at its resting potential, so that the cell A-induced voltage signals in cell B will be blocked (see Fig. 5 and Materials and Methods). We use this new multiple whole-cell patch electrode recording technique to study the photoreceptor coupled network in the salamander retina because this preparation has several advantages. (1) All retinal neurons are relatively large and they can be easily targeted under visual guidance for stable recordings (Wu, 1987). (2) Rods and cones are regularly spaced and form two-dimensional square lattices allowing easier spatial modeling (Attwell and Wilson, 1980; Attwell et al., 1984). (3) Previous pair recording results provided photoreceptor coupling parameters that can be extended for analysis of the multiple rod network: rod–rod coupling is ∼20 times stronger than rod–cone coupling and cones are not coupled (Attwell et al., 1984; Zhang and Wu, 2005), and thus it is an ideal preparation to study the rod coupling network. (4) Salamander rod and cone gap junctions are mediated by connexin36 (Zhang and Wu, 2004), the gap junction protein found in photoreceptors in most vertebrate species (Deans et al., 2002; O’Brien et al., 2012). Moreover, junctional conductance between salamander rods are linear and symmetrical (Zhang and Wu, 2005), similar to gap junction properties of photoreceptors in other animals (Hornstein et al., 2004; Li and Devries, 2004), and thus network properties learned from this project are likely to be applicable to photoreceptor systems in other species.

It has been suggested that many retinal diseases are mediated by abnormalities in retinal synaptic pathways. For example, rod and cone dystrophy in retinitis pigmentosa, macular degeneration, and several other diseases are related to photoreceptor dysfunction (Adler et al., 1999; Phelan and Bok, 2000; Pennesi et al., 2003, 2006; Abd-El-Barr et al., 2009). One hypothesis for rod–cone dystrophy (rod degeneration induces cone dystrophy) is that some “pathogenic molecules” leak from rods to cones via gap junction channels (the so-called bystander’s effect; Cusato et al., 2003b,a). Understanding spatiotemporal behaviors of signal spread in the photoreceptor coupled network will help to gain important insights on how photoreceptors function in healthy retinas and how they dysfunction in diseased states.

## Materials and Methods

### Preparations

Flat-mounted retinas of the larval tiger salamander (*Ambystoma tigrinum*, both sexes) were used in the proposed experiments (Werblin, 1978; Wu, 1987). These animals were purchased from Charles E. Sullivan and Kons Scientific and kept in aerated aquaria and fed with brine shrimp or dry fish food. Experimental procedures conformed to the ARVO Statement on the Use of Animals in Ophthalmic and Vision Research and the NIH guide for the Use of Laboratory Animals, and they were approved by the Committee of Animal Research of Baylor College of Medicine. Procedures for preparing retinal slices and flat-mounted retina have been described in previous publications (Wu et al., 2000; Zhang and Wu, 2009a), and retinal neurons as well as electrodes above the retina were visualized with an infrared camera (Olympus OLY150) attached to a Zeiss-DIC microscope.

### Electrophysiology

Simultaneous whole-cell in vitro recordings from up to eight neurons were obtained from flat-mounted isolated retinas as described previously (Attwell et al., 1984; Zhang and Wu 2009a). Electrode and rod positions were visualized with an IR camera via a 40× water immersion objective (n.a. = 0.75) of the recording microscope. We used patch recording pipettes (5–7 MΩ) filled with intracellular solution containing 120 mM potassium gluconate, 10 mM HEPES, 4 mM KCl, 4 mM MgATP, 0.3 mM Na_3_GTP, 10 mM sodium phosphocreatine and 0.5% biocytin, pH 7.25. We used two Quadro EPC 10 amplifiers for the experiments. A built-in LIH 8 + 8 interface board was used to perform simultaneous A/D and D/A conversion of current, voltage, command, and triggering signals from the amplifiers. The PatchMaster software and custom-written Matlab-based programs were used to operate two Quadro EPC 10 amplifiers and perform online and offline analysis of the electrophysiology data. The eight micromanipulators (Luigs & Neumann 8-Unit Micromanipulator System attached to eight head stages) were mounted on a ring specifically designed for our multipatching system. The eight micromanipulators were controlled by a remote 8-unit hydraulic devise, and tips of eight patch electrodes were first advanced together under the surface of the recording chamber solution then advanced separately under visual guidance via the objective to attach and break-in individual rods. Current steps were injected into the rods (±1 nA for 500 ms) to evoke postsynaptic potentials in adjacent rods. Since the dark membrane potentials of rods in the salamander retina are near −40 mV (Yang and Wu, 1997; Zhang and Wu, 2009a), the voltage signals were measured while the membrane potentials of all rods were held under current clamp at −40 ± 3 mV. For voltage-clamp block experiments, intermediate rods were held under voltage clamp at −40 mV (Wu and Yang, 1988; Yang and Wu, 1997; Zhang and Wu, 2009a, 2010).

### The infinite cable model

The rod coupled network is modeled with the linear infinite cable equation (Johnston and Wu, 1994) to determine whether current diffusion in the network is passive. We first modeled the rod coupled network with a two-dimensional lattice array [each rod is 10 µm in diameter and the inter-rod distance (soma diameter + telodendrion length) = 20 µm; Lasansky, 1973). Two next neighboring rods are connected with a junctional resistance *R*_j_ = 2,000 MΩ (junctional conductance *G*_j _= 500 pS; Zhang and Wu, 2005). We have shown that the indirect pathways have insignificant contributions to the rod–rod junctional conductance (Fig. 5). We then modeled the two-dimensional network with a one-dimensional infinite cable in one direction, as we lumped the junctional resistances in the orthogonal direction with the cell’s leak resistance that results in the cell’s membrane resistance *r*_m _= 250 MΩ·10 µm (soma diameter, 10 µm). The internal resistance *r*_i _= 2,000 MΩ/20 µm (cytosolic distance: soma diameter + telodendrion length = 20 µm; Lasansky, 1973). From Equations 3 and 4, the space constant $\lambda =\sqrt{r_{m}/r_{i\;}}=5\;\mu m$ and the time constant τ = *c*_m_*r*_m_ = 78 ms [where *c*_m_ = (1 µF/cm^2^) (2πa) = 0.0314 µF/µm]. Put all these parameters into the infinite linear cable equation (Johnston and Wu, 1994) and the analytic solutions are given below, and the parameters given above were used for simulations in Figure 2.

(1) $$\frac{1}{r_{i}}\frac{\partial^{2}V_{m}}{\partial x^{2}}=c_{m}\frac{\partial V_{m}}{\partial t}+\frac{V_{m}}{r_{m}},$$

where $V_{m}$ is the membrane potential (mV), $r_{i}$ is the axial resistance (Ω/cm), $r_{m}$ is the membrane resistance(Ω-cm), and $c_{m}$ is membrane capacitance (F/cm).

General solution to the equation:

(2) $$V_{m}(t,x)=\frac{r_{i}I_{0}\lambda }{4}\left[e^{-x/\lambda }erfc\left(\frac{x/\lambda }{2\surd t/\tau_{m}}\right)-e^{x/\lambda }erfc\left(\frac{x/\lambda }{2\surd t/\tau_{m}}\right)\right].$$

(Current step $I_{0}$ into rod00) space constant:

(3) $$\lambda =\sqrt{r_{m}/r_{i\;}}.$$

Time constant:

(4) $$\tau_{m}=c_{m}r_{m\;}.$$

Steady-state solution (t→∞):

(5) $$V_{m\;}(\infty ,x)=\frac{r_{i}I_{0}\lambda }{2}e^{-x/\lambda }.$$

Transient-state solution at *x = *0:

(6) $$V_{m\;}(t,0)=\frac{r_{i}I_{0}\lambda }{2}erfc\left(\sqrt{t/\tau_{m}}\right).$$

Conduction velocity:

(7) $$\emptyset =\frac{dx\;}{dt}=\frac{2\lambda }{\tau_{m}}.$$

### Statistics

All data are presented in figures and the table with means and standard deviations. Student’s unpaired *t* test was used to test the significance (with *p* values) of differences between various sets of responses and between measured and calculated values.

## Results

### Spatiotemporal profiles of the rod coupled network

**Figure 1.**
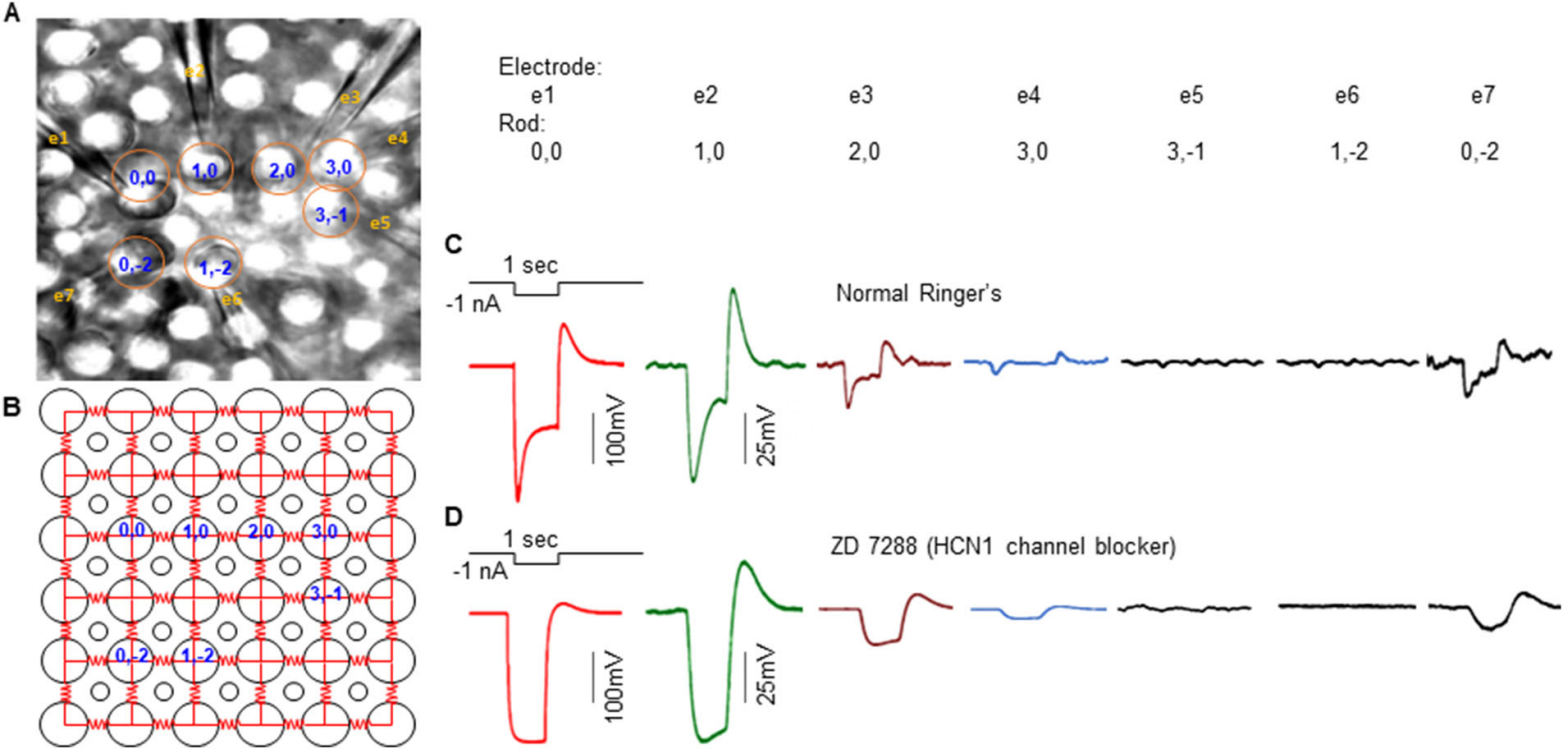
***A***, Locations of seven rods recorded in flat-mounted salamander retina with the multiple patch electrode recording system. RodXY are rod locations in the 2D array and e1–e7 are electrode numbers. ***B***, Original locations of the seven rods before recording, as rods were slightly pushed from original positions (yellow circles) by the patch electrodes. Large circles, rods; small circles, cones. Red resistors are coupling resistance between rods (coupling resistance between rods and cones are much higher and thus not shown). ***C***, Voltage responses of Rods1,0; 2,0; 3,0; 3,−1; 1,−2; and 0,−2 to a −1 nA current step (1 s) injected into Rod0,0. ***D***, Repeating experiments in ***C*** in the presence of 100 µM ZD7288.

**Figure 2.**
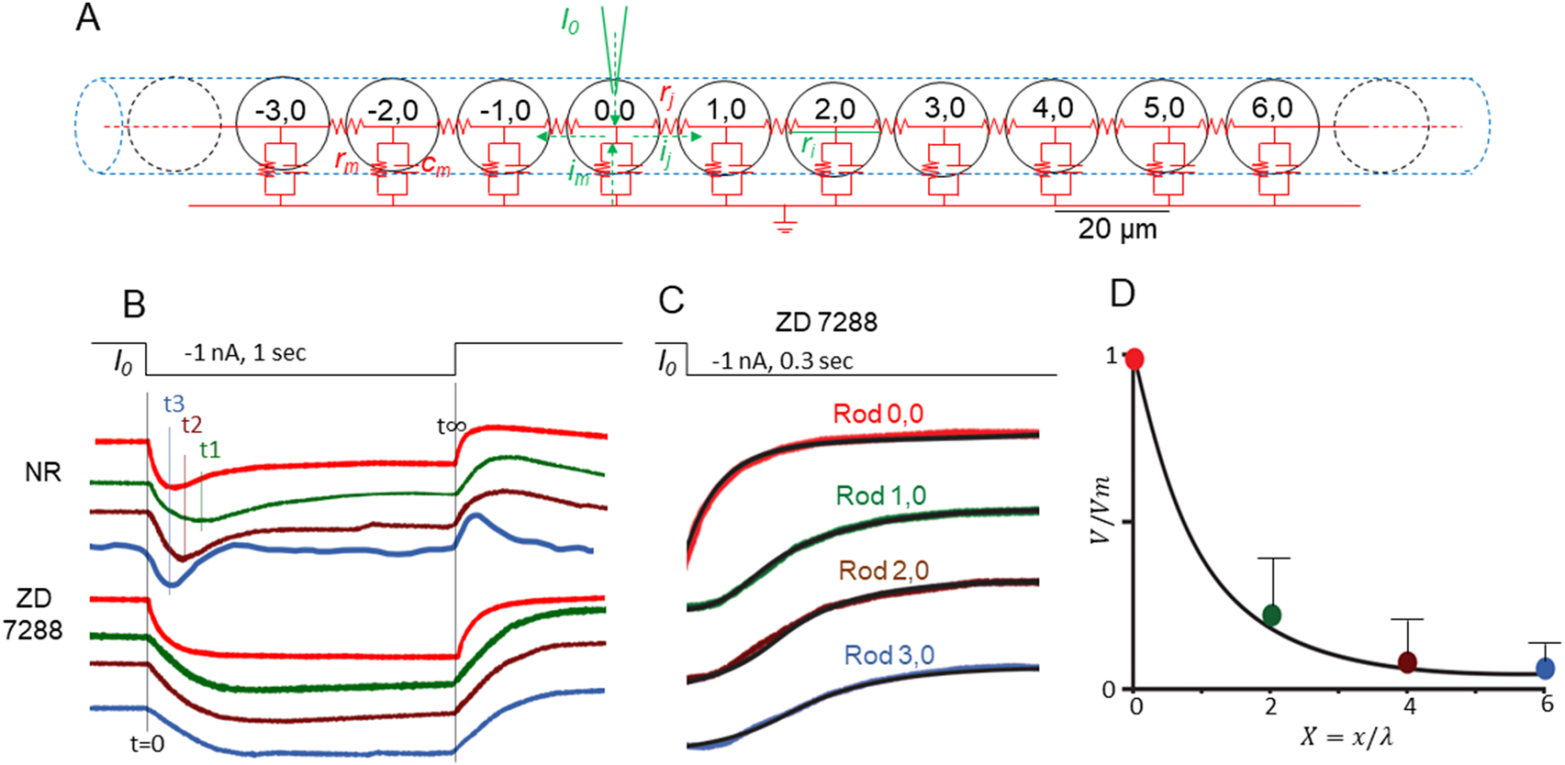
***A***, Schematic diagram of infinite linear cable representation of the rod coupled network. We reduced the two-dimensional lattice into a one-dimensional (*x* direction) cable by lumping coupling resistance in the *y* direction with the rod’s leak resistance, resulting in a membrane resistance *r*_m _= 250 MΩ·10 µm. The internal resistance is the cytoplasmic resistance between two adjacent rods (20 µm apart) *r*_i _= *R*_j_/20 µm = 2,000 MΩ/20 µm (Zhang and Wu, 2005, 2009b). These yield a space constant λ = 5 µm and time constant τ = 78.5 ms. ***B***, Normalized responses of Rod0,0 (red), 1,0 (green), 2,0 (brown), and 3,0 (blue) in the absence (top 4 curves) and presence of ZD (bottom 4 curves). The time of response peak of Rod1,0; 2,0; and 3,0 in the absence of ZD are shown as t1, t2, and t3, respectively. ***C***, In the presence of ZD, we replotted the onset charge curves in B (inverted display) and found that they agree with the predictions of the infinite cable equation (complementary error function solutions, black curves in the colored traces; Equation 6 in Materials and Methods, The infinite cable model; Johnston and Wu, 1994). ***D***, Average (*n* = 5) steady-state response amplitudes (with standard deviations as error bars) of Rod0,0; 1,0; 2,0; and 3,0 in ZD normalized against the amplitude of Rod0,0 (colored dots) versus distance in the cable (*X* = *x*/λ), and they agree with the steady-state solution of the cable equation with a space constant 5 µm (black solid curve; Eq. 5 in Materials and Methods, The infinite cable model). Error bars are standard deviations.

Figure 1 illustrates voltage responses of six postsynaptic rods to current injection into one rod in a flat-mounted salamander retina recorded with the multielectrode whole-cell recording system. Voltage responses of Rod1,0; 2,0; 3,0; 3,−1; 1,−2; and 0,−2 (locations shown in Fig. 1*A*,*B*) to a −1 nA current step (1 s) into Rod0,0 are shown in Figure 1*C*. We repeated these experiments on the same set of rods in the presence of 4-ethylphenylamino-1,2-dimethyl-6-methylaminopyrimidinum chloride (ZD7288), an HCN1 channel blocker (Barrow and Wu, 2009), and results are shown in Figure 1*D*. Since Rod0,0; 2,0; and 3,0 are in a linear array, we model the array as an infinite linear cable as shown in Figure 2*A* (details are described in Materials and Methods, The infinite cable model). Figure 2*B* shows the normalized charge curves (onset time courses) and steady-state decay time courses of these rods in the absence and presence of ZD. In the presence of ZD, we replotted in Figure 2*C* the onset charge curves (inverted display, colored traces) and found that they agree with the predictions of the infinite cable equation (complementary error function solutions, dark black curves; Equation 6 in Materials ad Methods; Johnston and Wu, 1994). We recorded another four sets of rods in linear arrays, and they all showed similar response patterns with charge curves following the time course of Equation 6 in the linear cable model. Moreover, the spatial decay of steady-state response amplitudes of the rod array in ZD are plotted in Figure 2*D*, with a normalized (over five linear rod arrays) curve that follows the prediction of the linear cable in Equation 5 with a space constant 5 µm (see Materials and Methods). These results suggest that in the absence of *I*_h_, the rod coupled network behaves like linear infinite cables in which electrical signals spread passively via gap junctions. From Equation 7 in the infinite cable model, the conduction velocity of signal spread in the coupled rod network in the absence of HCN channels is Φ = 2λ/τ_m _= 2 × 5 µm/80 ms = 125 µm/s.

Under physiological conditions (in the presence of *I*_h_), however, the passive rod network is perturbed by a nonlinear current *I*_h_ that shapes the amplitude and kinetics of signal spread in the network. When rods are hyperpolarized by either light or negative current steps, *I*_h_ is activated that results in a time-dependent depolarization and a sag at the onset and a transient overshoot at the offset of the steps (Attwell and Wilson, 1980;Attwell et al., 1984). *I*_h_ activation truncates the passive signal spread and results in peak amplitudes that deviate from the predictions of the infinite cable equation. The time of response peak of Rod1,0; 2,0; and 3,0 in normal Ringer’s are shown as t1, t2, and t3, respectively, in Figure 2*B*, top portion, indicating that the voltage responses peak earlier in distant rods. Values of t1, t2, and t3 of the five sets of rods in linear arrays are given in Table 1, with an average ± SD t1 value of 184 ± 11 ms, t2 value of 130 ± 10 ms, and t3 value of 75.6 ± 5.5 ms. From time-to-peak values against distance, we obtained a negative conduction velocity of peak response: Φp = −10 µm / (184–130)ms = −185 µm/s. This is the first time that the “inductive” behavior of the photoreceptor coupled network proposed by Detwiler and Hodgkin (Detwiler et al., 1978, 1980) has been demonstrated by time-to-peaks of current-evoked voltage spread in multiple rods recorded simultaneously at various distances*.* The negative conduction velocity value obtained here is of the same order of magnitude as the conduction velocity of peak responses of a turtle cone to a narrow light bar at various distances from the recorded cone (mean Φp = −260 µm/s; Detwiler et al., 1978, 1980).

**Table 1.** Time-to-peak of Rod1,0 (t1), Rod2,0 (t2), and Rod3,0 (t3), orientation (degree from the nasal–temporal axis) and the maximum voltage response in Rod0,0 of five linear arrays of 4–7 rods

Linear array	t1(ms)	t2(ms)	t3(ms)	Orientation (°)	*V*_max_ (mV)
1	174	123	72.5	75	120
2	185	131	75.2	130	105
3	170	121	70.8	110	110
4	201	149	86.2	20	130
5	192	129	73.4	40	125
Average ± SD	184 ± 11	130 ± 10	75.6 ± 5.5		

### Current flows in a voltage-clamped rod coupled network follow Ohm’s law

**Figure 3.**
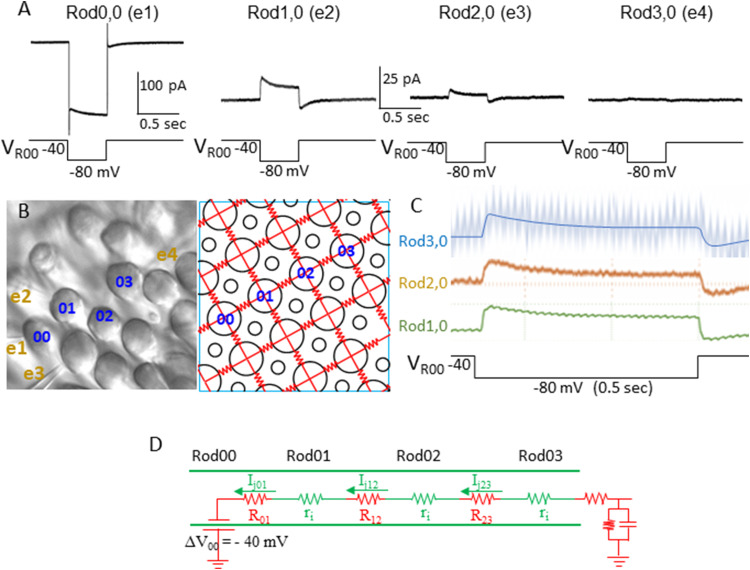
***A***, Current responses of four rods in a linear array (rod locations are shown in ***B***) to a voltage-clamp step (−40 to −80 mV, 0.5 s) in Rod0,0. Rod1,0; Rod2,0; and Rod3,0 were voltage clamped at −40 mV sequentially and the peak values of *I*_j01_ and *I_j_*_02_ agree with the prediction of the Ohm’s law (see text for statistics). ***C***, Normalized current responses of Rod1,0; Rod2,0; and Rod3,0 exhibited identical waveforms, suggesting that current flow within the coupled array under voltage clamp is linear. ***D***, schematic diagram of internal resistance (*r*_i_), coupling resistance (*R*_j_), and junctional currents (*I*_j_) in the four coupled rods.

When a linear array of coupled rods (Fig. 2*A*) is voltage clamped at their resting potential (−40 mV), a voltage step in one rod (Δ*V_R_*_00_ = −80 mV − −40 mV = −40 mV) induces current flows in the next neighboring rod (Rod1,0) through the gap junction. This junctional current (*I*_j01_) equals the quotient of the driving force (Δ*V_R_*_00_) and the resistance of the current pathway (ΔV/R, Ohm’s law). Thus the junctional current *I*_j01_ in rod1,0 generated by a voltage step of −40 mV in Rod0,0 is *I*_j01_=Δ*V*/*R* = −(−40 mV) / *R*_j _= 40 mV / 2,000 MΩ = 20 pA. For Rod2,0, the resistance of the current pathway must include two *R*_j_ values (*R*_j_ is the junctional resistance = 1/*G*_j_) and one internal resistance of the cable *r*_i _= 2,000 MΩ/20 µm (see Materials and Methods, The infinite cable model). However, since Rod1,0 was not voltage clamped (to avoid voltage-clamp block, see Fig. 5), *V*_R10_ was free-floating allowing *I*_j02_ flowing across it with the driving force *V*_R00_ – *V*_R20_=−80 mV − (−40 mV) = −40 mV. Therefore, *I*_j02_ can be approximately estimated (due to the uncertain *V*_R10_ value and possible current leak in Rod1,0) as *(V*_R00_ – *V*_R20_) / (*R*_j _+ *R*_j _+ *r*_i_) = −(−40 mV) / (2,000 + 2,000 + 2,000)MΩ = 6.7 pA. For Rod3,0, since both Rod1,0 and Rod2,0 were not voltage clamped, the uncertainties are more significant and thus the amplitude of *I_j_*_03_ was not determined. In four linear arrays, we found that the average amplitudes of *I*_j01_ and *I*_j02_ are close to the values of the Ohm’s law-based calculations: *I*_j01_*_ _*= 17 ± 4.5 pA; *I*_j02_ = 6.4 ± 2.3 pA. To compare these values with the calculated values, 20 and 6.7 pA, Student’s unpaired *t* test yields *p* values of 0.2399 and 0.9005, indicative of insignificant statistical differences. Moreover, in contrast to the voltage responses of the rod coupled network, the junctional current waveform does not change with distance (Fig. 3*C*). A schematic diagram of internal resistance (*r*_i_), coupling resistance (*R*), and junctional currents (*I*_j_) in the four coupled rods under voltage clamp is given in Figure 3*D*. These results suggest that the current flow within the rod coupled network is linear and obeys Ohm’s law when the membrane voltage is clamped. Nonlinear behaviors of the rod network, such as the negative conduction velocity of peak voltage responses described in the last section, are mediated by membrane voltage- and time-dependent mechanisms.

### Spatial symmetry of signal spread in the rod coupled network

**Figure 4.**
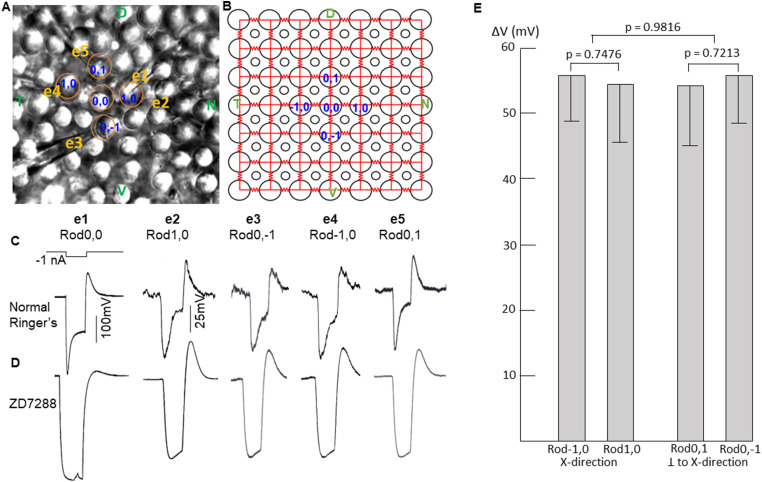
***A***, Five rods recorded in flat-mounted salamander retina with the multiple patch electrode recording system. ***B***, Original locations of the five rods before recording. ***C***, Voltage responses of Rod1,0; Rod0,−1; Rod−1,0; and Rod0,1 to a −1 nA current step (1 s) injected into Rod0,0. ***D***, Same experiments as in ***C*** in the presence of 100 µM ZD7288. ***E***, Average voltage responses of eight five-rod clusters to −1 nA current injection into the center rod. Rod−1,0/Rod1,0 and Rod0,1/Rod0,−1 are in two orthogonal directions. Error bars are standard deviations, and *p* values from *t* test indicate that there are no statistical significant differences between Rod−1,0 and Rod1,0 responses, between Rod0,1 and Rod0,−1 responses, and between the two orthogonal rod pairs.

To determine whether rod–rod coupling is symmetrical in the two-dimensional network, we recorded rods in five-rod clusters (Fig. 4*A*) and measured voltage responses of four adjacent rods to current injection into the center rod. Figure 4*C* shows the voltage responses of two adjacent rods in one direction are about the same as those in the orthogonal direction (the input resistance of the four rods are nearly identical; Zhang and Wu, 2005). This spatial symmetry was maintained in the presence of ZD (Fig. 4*D*), suggesting that the passive spread of signals (coupling coefficient) is nearly the same in one direction as the other. We performed these experiments in eight five-rod clusters and found that the average responses in rods in two orthogonal directions to current injection into the center rod are not significantly different. In Figure 4*E*, we plotted the average voltage responses (±SD) of eight five-rod clusters to −1 nA current injection into the center rod. Rod−1,0/Rod1,0 and Rod0,1/Rod0,−1 are in two orthogonal directions. *p* values from the *t* test indicate that there are no statistical significant differences between Rod−1,0 and Rod1,0 responses, between Rod0,1 and Rod0,−1 responses, and between the two orthogonal rod pairs. These results suggest that the signal spread in the coupled rod network is symmetrical in the two-dimensional rod network, consistent with our observation that the time-to-peak and the conduction velocity of peak responses are independent of the orientation of the linear arrays (Table 1).

### Effects of voltage-clamp block of intermediate rods in the network

**Figure 5.**
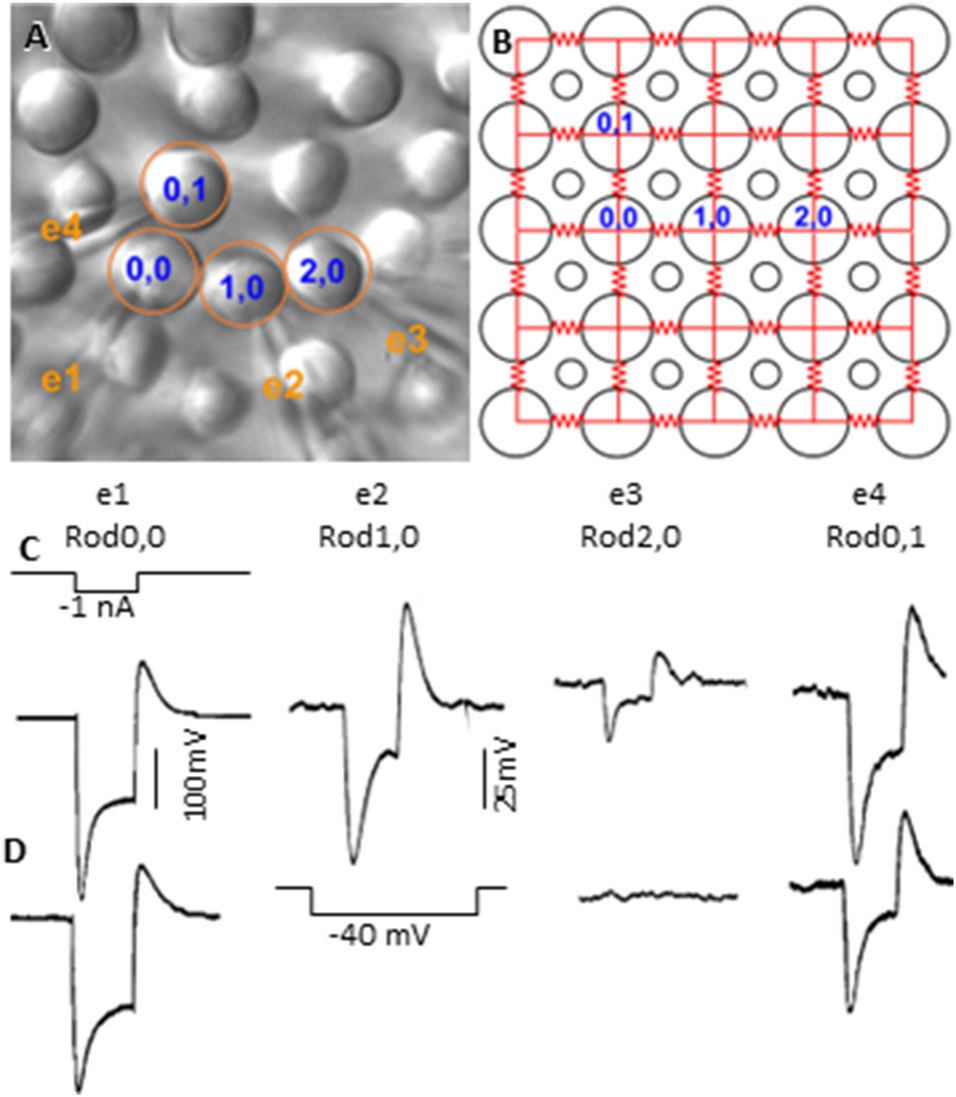
***A***, Four rods recorded in flat-mounted salamander retina with the multiple electrode recording system. ***B***, Original locations of the four rods before recording. ***C***, Voltage responses of Rod1,0; Rod0,1; Rod0,2; and Rod0,1 to a −1 nA current step (1 s) injected into Rod0,0. ***D***, Same experiments as in ***C*** except that Rod0,1 was voltage clamped at −40 mV.

Figure 5 shows that in a linear array of Rod0,0; 1,0; and 2,0, voltage clamping Rod1,0 blocks the voltage response of Rod2,0 to current injection into Rod0,0. We obtained similar results from nine three-rod linear arrays. We also recorded voltage responses of rods in various multirod arrays where voltage clamping intermediate rods at −40 mV (resting potential), to determine how blockade of signal transmission of photoreceptors in any position(s) affects the rod network. For example, we examined the effect of voltage-clamp block of Rod1,0 and Rod0,1 on the voltage response of Rod1,1 to current injection into Rod0,0. Assuming that the coupling coefficient of two next neighbor rods is α (∼0.2; Zhang and Wu, 2005), then the peak response of Rod1,1 would be 2α^2^*V*, where *V* is the peak voltage change of Rod0,0 elicited by current injection into it. We found that voltage-clamp blocking Rod1,0 or Rod0,1 leads Rod1,1 response to be α^2^*V*. This suggests that the Rod0,0–Rod1,0–Rod1,1 and the Rod00–Rod0,1–Rod1,1 coupling pathways are symmetrical. When both Rod1,0 and Rod0,1 were blocked, Rod1,1 had no response to current injection into Rod0,0. In Figure 5, voltage clamping Rod1,0 also reduced the response of Rod0,1 (occurred in three out of the nine three-rod arrays), suggesting in some cases that indirect coupling pathways contribute to rod coupling signals, possibly due to larger α values between certain rods. It is worth to note that even in the case of larger α between some rods, voltage clamping Rod1,0 still completely blocked coupling signals from Rod0,0 to Rod2,0.

## Discussion

In this study, we investigate the network properties of two-dimensional rod coupled arrays in the vertebrate retina by using the newly available multiple patch electrode recording system and to determine the simultaneous responses and synaptic connectivity of up to eight retinal neurons. We found that current-evoked voltage responses of rods recorded simultaneously in a linear array are highly nonlinear, with shorter time-to-peak in distant rods, similar to an observation obtained by comparing responses of a turtle cone to light stimuli at various distances from the recorded cell (Detwiler et al., 1978, 1980; Detwiler and Hodgkin, 1979). We also found that when the array of rods was voltage clamped, this nonlinear behavior disappeared, and the current flow in the coupled rod network obeyed Ohm’s law, suggesting that the nonlinearity is mediated by a voltage-dependent mechanism. We thus studied the current-evoked responses of the rod array in the presence of *I*_h_ (mediated by HCN channels) blocker ZD7288 and in which the voltage signal spread in the rod coupled network is passive and it follows the linear infinite cable equation. This suggests that without the voltage- and time-dependent *I*_h_, the rod coupled network behaves linearly and voltage signals diffuse passively within the coupled rod network.

The linear infinite cable model suggests that the conduction velocity of voltage signal spread in the coupled rod network in the absence of *I*_h_ is Φ = 2λ/τ*_m_*_ _= 2 × 10 µm/80 ms = 125 µm/sec. In the presence of *I*_h_, on the other hand, the conduction velocity of the current-evoked peak response, Φp = −10 µm/0.04s = −185 µm/sec, a value of the same order of magnitude of the conduction velocity of peak responses of the turtle cone to a narrow light bar delivered at various distances from the recorded cone (mean value, 260 µm/s; Detwiler et al., 1978, 1980). Such negative conduction velocity is a characteristic feature of an inductive circuit in which current flows in two opposite directions (Detwiler et al., 1978, 1980; Detwiler and Hodgkin, 1979; Halliday et al., 2001), as the injected current is inward and *I*_h_ is outward (Barrow and Wu, 2009). In rods, the delayed conductance changes associated with *I*_h_ mimic an inductance and make the network like a high-pass filter with series resistance and parallel inductance (Detwiler et al., 1980). Moreover, photocurrent-elicited hyperpolarizing voltage signals in rods have a rise time which is comparable with the time constant of *I*_h_-mediated depolarizing voltage signals (Attwell and Wilson, 1980; Attwell et al., 1985). As the rise time of the hyperpolarizing voltage signal increases with distance, it becomes increasingly close to the time constant of *I*_h_ (Attwell and Wilson, 1980; Barrow and Wu, 2009), and thus *I*_h_ truncates the hyperpolarizing voltage signal at earlier times.

By measuring voltage responses of adjacent rod pairs in orthogonal directions, we found that rod–rod coupling is largely symmetrical in the two-dimensional rod coupled network. Voltage-clamp block of one next neighboring rod suppresses signal spread into the second neighboring rod but not diagonal rods, suggesting that current spread from one rod to a second neighboring rod is primarily mediated via the next neighboring rod between them.

Our results demonstrate that *I*_h_ is the main voltage- and time-dependent mechanism that shapes signal spread in the network. It has been shown, however, that voltage-gated calcium currents also exist in photoreceptors (Bader et al., 1979), and previous works have suggested that the calcium current activation range is more positive than the dark membrane potential (Bader et al., 1979; Attwell et al., 1987). Therefore, analyzing voltage responses of the coupled rod network elicited by negative current injections (Figs. 1, 2) or by light could largely avoid calcium current influence on voltage signal spread in the rod network. The only response component where calcium current may be observable is the transient depolarization at the offset of the current step. In ZD, the depolarizing off responses were mediated primarily by anode-break depolarization-activated calcium current. In control, the depolarizing off responses were mediated by such calcium current as well as the deactivation of *I*_h_.

It is possible that rod–cone coupling contributes to signal spread in the rod network. Previous studies have shown that in the salamander retina, rod–cone coupling is ∼25 times weaker than rod–rod coupling [Attwell et al., 1984; in contrast to the recent finding that in the mouse retina, rod–cone coupling is much stronger than rod–rod coupling (Jin et al., 2020)]. Therefore rod–cone coupling plays a minor role in mediating voltage signal transmission in the coupled rod network. In the infinite cable model, we lump rod–cone gap junction resistance with the membrane resistance of the rods (*r*_m_), and our data in Figure 2 show that the model agrees with the measurements reasonably well.

It has been shown that the input–output relations of the photoreceptor→second-order cell synapses are nonlinear with the highest slope gain near the dark potentials (Attwell et al., 1985). Therefore, spread the light-evoked signal in a rod, for example, to adjacent rods results in smaller signals in multiple presynaptic rods. These small rod signals converge to the postsynaptic BCs/HCs with higher voltage gains and less noise (signal averaging), and the sum of the postsynaptic signals is significantly larger than that transmitted via a single rod output synapse (Wu, 1991).

A functional advantage of the *I*_h_-mediated network high-pass filter and response time-to-peak shortening of distant rods is that it helps the integration of rod responses in second-order cells, such as bipolar cells. It may take signals from distant rods more time to reach the bipolar cell soma because of the longer dendritic processes. Shortening the response time-to-peak of distant rods helps to synchronize the integration time of bipolar cell peak responses and improves the spatial and temporal resolution of the synaptic output of the rod network (Detwiler et al., 1978).
